# Quantitative Study of the Weakening Effect of Drilling on the Physical and Mechanical Properties of Coal–Rock Materials

**DOI:** 10.3390/ma16196424

**Published:** 2023-09-27

**Authors:** Lidong Yin, Yin Zhang, Lianpeng Dai, Jiping Zhang, Jiajun Li, Chenchen Yang

**Affiliations:** 1 School of Mechanics and Engineering, Liaoning Technical University, Fuxin 123000, China; 2 School of Environment, Liaoning University, Shenyang 110036, China

**Keywords:** high strain rate, SHPB, drilled coal specimens, crack extension, energy dissipation

## Abstract

Coal seam drilling is a simple, economical, and effective measure commonly used to prevent and control rock burst. Following rock burst, coal exhibits significant dynamic characteristics under high strain-rate loading. Our purpose was to determine the physical processes associated with impact damage to drilled coal rock, and its mitigation mechanism. An impact test was carried out on prefabricated borehole coal specimens, and the impulse signals of the incident and transmission rods were monitored. The crack initiation, expansion, and penetration of coal specimens were video-recorded to determine the mechanical properties, crack expansion, damage modes, fragmentation, and energy dissipation characteristics of coal specimens containing different boreholes. The dynamic compressive strength of the coal specimens was significantly weakened by boreholes under high strain-rate loading; the dynamic compressive strength and the dynamic modulus of elasticity of coal rock showed a decreasing trend, with increasing numbers of boreholes and a rising and decreasing trend with increasing borehole spacing; the number and spacing of boreholes appeared to be design parameters that could weaken coal–rock material under high strain-rate loading; during the loading of coal and rock, initial cracks appeared and expanded in the tensile stress zone of the borehole side, while secondary cracks, which appeared perpendicular to the main crack, expanded and connected, destroying the specimen. As the number of boreholes increased, the fractal dimension (D) and transmission energy decreased, while the reflection energy increased. As the borehole spacing was increased, D decreased while the reflective energy ratio decreased and increased, and the transmissive energy ratio increased and decreased. Drilling under high strain modifies the mechanical properties of impact damaged coal rock.

## 1. Introduction

Rock burst is a world-class problem recognized by the mining and rock mechanics communities at home and abroad, and is one of the most serious dynamic disasters in coal mines [1,2,3]. Currently, China’s shallow high-quality coal resources are exhausted, and coal mining is gradually shifting to the deep part [4]. The deep coal rock is in a complex mechanical environment of high ground stress, high osmotic pressure, high temperature, and engineering perturbation, whose mechanical properties are different from those of shallow coal rock, and are susceptible to dynamic disasters such as rock burst, explosion of methane and coal dust, etc. With the increase in deep mining, the frequency and intensity of the occurrence of rock burst have been gradually increasing, which is one of the world’s underground engineering problems; it poses a serious threat to the safety of personnel and equipment, as well as the mining efficiency of the mines. The impact damage of coal rock after the initiation of rock burst is an obvious kinetic damage process with significant kinetic characteristics under high strain-rate loading, while the mechanical response of the roadway surrounding rock to the effect of high strain-rate loading determines the intensity of the impact damage, so it is particularly important to unload the deep elastic surrounding rock to reduce the destructive intensity of the impact. The core concept of pressure relief is to take measures to release the transferred stress in advance [5]. As a simple, economical, and effective method to prevent the impact of ground pressure, roadway boreholes have been widely used, which can reduce the stress state of surrounding rock and change the stress distribution of surrounding rock. The pressure relief zone formed after the borehole is broken has a weakening effect on the propagation of stress waves. Therefore, studying the mechanical properties of borehole-modified coal rock under a high strain rate can provide a reference for the stability of underground roadway surrounding rock.

Currently, many scholars have conducted a series of studies on the mechanical properties of coal and rock under high strain rates. Santosh Kumar Ray et al. [6] reported a list of mine explosion disasters in the last two decades, a critical review of the different prevention and constructive measures, and its recent development to avoid firedamp and coal dust explosions. Igor Ivanovich Bosikov et al. [7] developed a new methodology for modeling the ventilation network parameters of the mine to increase the reliability of controlling the aerogas mode at the excavation site. Mahdi Saadati et al. [8] used Hopkinson rods for spall testing to study the dynamic tensile behavior of granite samples containing preformed cracks. Selahattin Akdag et al. [9] performed dynamic Type I fracture toughness tests on cracked V-notched semicircular bent granite specimens using a split Hopkinson pressure bar (SHPB) to investigate the relationship between the strain burst mechanism and its dynamic fracture extension. Payam Asadi et al. [10] explored the effects of existing defects or notches on the tensile strength of synthetic rocks under static and dynamic loading. Mehrdad Imani et al. [11] investigated the dynamic characteristics and failure mechanisms of Brazilian disc specimens under high-speed loading, using a two-dimensional particle flow code (PFC2D) to simulate the Brazilian disc samples, and the results of the numerical model were in good agreement with the experimental measurements. Diouri, K et al. [12] investigated the dynamic behavior of asphalt mortar at high strain rates of 281.7–830.3 s^- 1^ by means of the split Hopkinson pressure bar (SHPB) test, and analyzed the effects of temperature and long-term oven aging on the dynamic properties of asphalt mortar under impact loading.

Scholars have conducted a large number of theoretical and practical studies on coal borehole measures and achieved more results. Sachin Kumar et al. [13] investigated the effect of defect inclination on the mechanical behavior of rock samples containing preexisting defects under dynamic loading conditions with different filling conditions. Wang Aiwen et al. [14] conducted uniaxial loading tests on prefabricated borehole coal specimens to study the changing law of impact propensity of borehole coal specimens; they introduced the fractal dimension of crushed particles and the new surface area, and analyzed the energy dissipation law in the crushing process of borehole coal specimens. Jia Chuanyang et al. [15] studied the influence of hole diameter, hole spacing, and hole depth on the strength of prefabricated hole specimens through indoor tests, and analyzed the damage pattern of specimens under the influence of different parameters. Shuang Haiqing et al. [16] studied the effects of different borehole inclination angles on the stress–strain curve, strength characteristics, damage pattern, and damage degree of coal under uniaxial compression and steady pressure conditions. 

In this research, SHPB impact tests were conducted on coal specimens with different borehole schemes to study the dynamic mechanical properties of borehole-modified coal specimens under high strain rates. The monitoring was carried out using high-speed cameras to analyze the crack extension, crushing law, and energy characteristics of the coal specimens, in order to probe deeply into the influence of the borehole arrangement on the effect of pressure unloading.

## 2. Materials and Methods

### 2.1. Specimen Preparation

**Figure 1:**
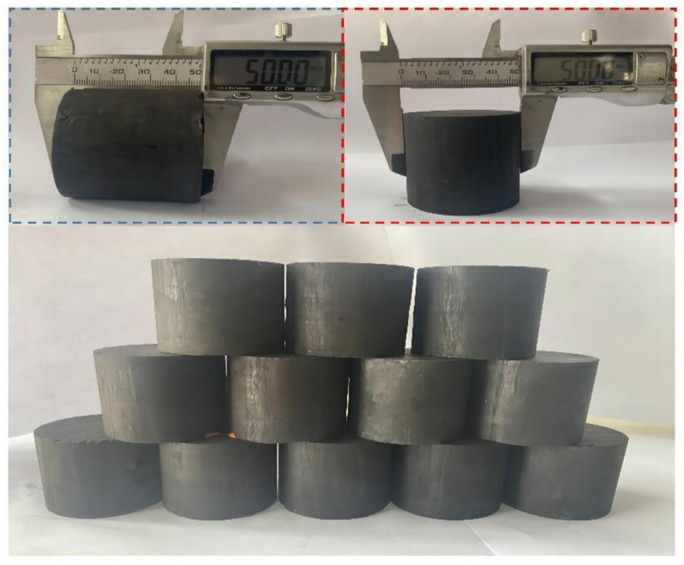
Physical images of coal specimens.

The specimens were taken from a mine in the northwestern part of Changwu County, Xianyang, Shaanxi Province, China, with a production capacity of 4.5 Mt/a. The average depth of the working face is about 990 m, the average thickness of the coal seam is 7.8 m, the inclination angle of the seam is 0–8°, and the average inclination angle is 4°. There are no faults, trap columns, or magma intrusions, and the structure is simple in the working face. In accordance with the recommended standards of the International Society for Rock Mechanics (ISRM), the coal specimens were cored, polished, and processed into cylindrical coal specimens with a diameter of 50 mm and a height of 50 mm, and drilled according to the test program, which required the borehole direction to be perpendicular to the direction of the joints. Next, the two ends of the specimens were polished, so that the flatness of the two end surfaces and the perpendicularity error were within 0.02 mm. Physical drawings of the processed coal specimens are shown (Figure 1).

**Figure 2:**
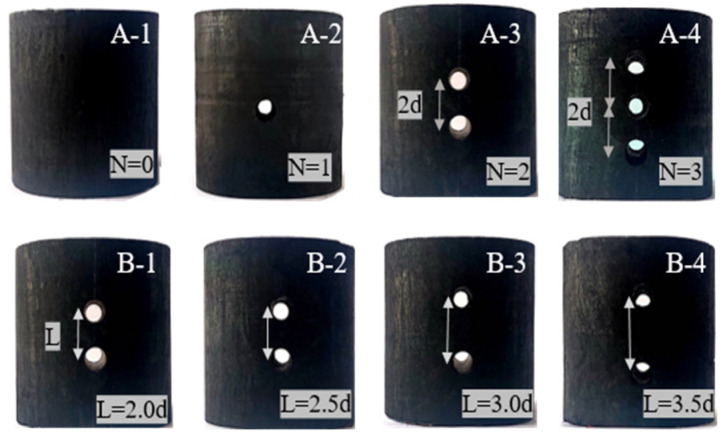
Specimens with different borehole schemes.

To reduce errors that may be caused by the non-uniformity of coal, six parallel specimens were prepared for each group. The data showing large dispersions were removed, and three groups of valid data were selected. The arrangement of boreholes is shown (Figure 2), with the diameter (d) of the boreholes being adjusted to d = 6 mm. A-1~A-4shows four groups of specimens with a variable number (N) of boreholes, where N = 0, 1, 2, 3; and B-1~B-4 shows four groups of two-borehole coal specimens with variable borehole core spacing (L), where L = 2.0, 2.5, 3.0, and 3.5d.

### 2.2. Test System

**Figure 3:**
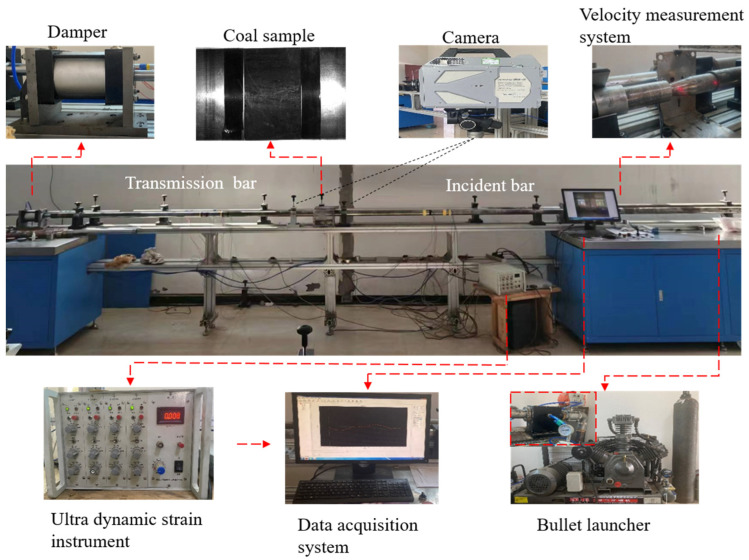
SHPB test system diagram.

This test used an SHPB device with a bar diameter of 50 mm for impact testing (Figure 3). The device that was used consisted mainly of a spindle-shaped bullet, an incident bar, and a transmitted bar, with bar lengths of 2500 mm in high hard aluminum with a longitudinal wave of 5000 m/s and a modulus of elasticity of 71 GPa. To control the speed of impact, the air pressure was adjusted to deliver a half-sinusoidal wave loaded with a constant high strain rate (110 s^−1^) to the coal specimens. Two sets of strain gauges, which were connected to the incident and transmitted bars, were placed 1250 mm away from the location where the coal specimens were placed. The impulse signals generated by the impact were acquired using an ultra-dynamic strain instrument and an oscilloscope, and were recorded and saved. During the loading process, an ultra-high-speed camera from Japan’s PHOTRON LIMITED with a resolution of 640 × 280 and a frame rate of 100,000 fps/s was used to record the damage pattern of the coal specimens.

The equations for the dynamic stress, strain, and average strain rate of the coal specimens can be derived according to one-dimensional stress wave theory and the assumption of stress uniformity [17,18,19] as follows:

(1) $$\left\{\begin{array}{l} \sigma =\frac{EA}{A_{0}}\varepsilon_{t}\\ \varepsilon =-\frac{2c_{0}}{l_{0}}\int_{0}^{t}\varepsilon_{r}dt\\ \dot{\varepsilon }=-\frac{2c_{0}}{l_{0}}\varepsilon_{r} \end{array}\right.$$

where *c*_0_ is the elastic wave velocity of the bars (5000 m/s); *E* is the elastic modulus of the bars (71 GPa); *A* is the cross-sectional area of the bars; and *A*_0_ and *l*_0_ are the original cross-sectional areas and lengths of the coal specimens, respectively.

### 2.3. Test Procedure 

Before conducting tests, the SHPB system was subjected to an impact test to ensure the strength of the connection between the bars and the stability of stress wave transmission. After uniformly applying petroleum jelly to reduce the impact of friction at the top, the two ends of the coal specimens were connected to the bars. Transparent acrylic baffles were added around the coal specimens to prevent splashing. After the coal specimens were installed, the ultra-high-speed camera was connected to track the destruction of the coal specimens, following which the bullet was adjusted to a predetermined position using the marked pusher. The launching air pressure was adjusted to achieve a predetermined impact speed, and upon completion of the preparation, the switch was turned on to conduct the impact test. 

### 2.4. Validity Analysis

**Figure 4:**
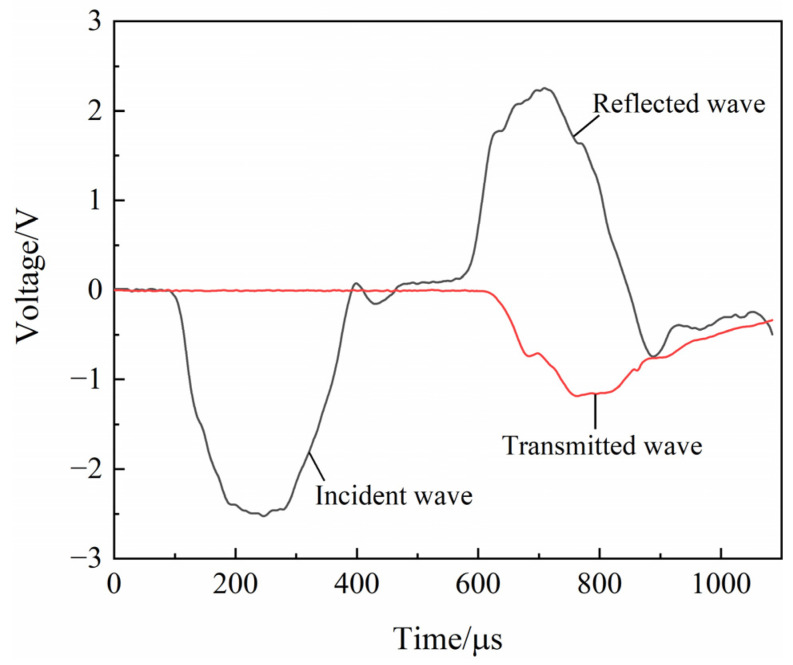
Typical waveform of coal specimen SHPB test.

**Figure 5:**
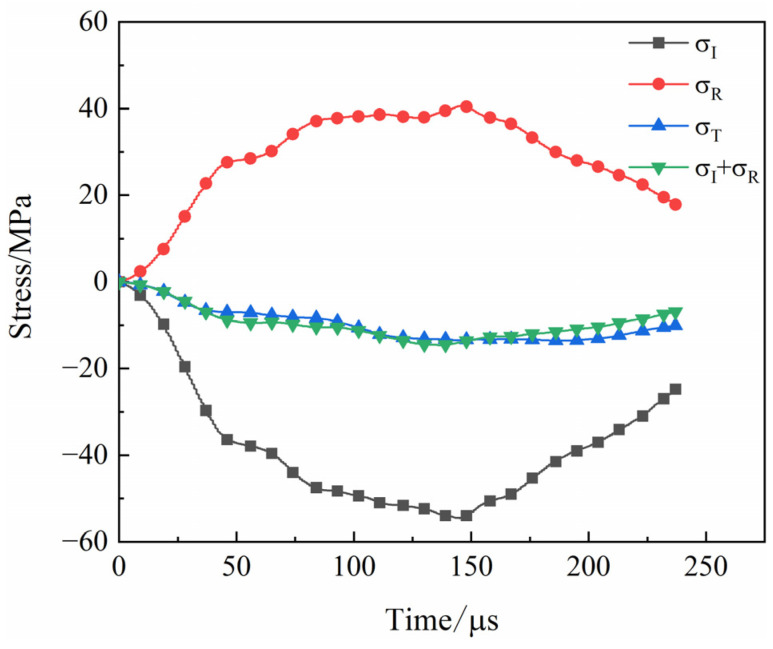
Dynamic stress balance verification.

The waveforms of the SHPB tests of the coal specimens are shown (Figure 4). The stress wave curves were smoother without obvious spikes [20]. During impact tests, stress balance verification is required to ensure the accuracy of test results [21]. The signals were recorded using an ultra-dynamic strain instrument, and the stress wave graphs were obtained via data processing (Figure 5), where *σ*_I_, *σ*_R_, and *σ*_T_ are the incident wave, reflected wave, and transmitted wave, respectively. The figure shows that the superposition curve of the incident and reflected waves was coincident with the curve of the transmitted wave, indicating that the stresses at both ends of the coal specimens had reached equilibrium during the loading process.

## 3. Results 

### 3.1. Stress–Strain Curve Analysis 

**Figure 6:**
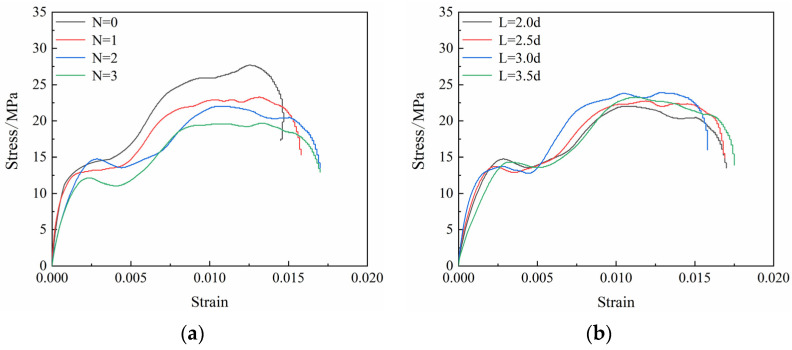
Stress–strain curves of specimens with (**a**) different numbers of boreholes and (**b**) different spacings of boreholes.

The stress–strain curves of the coal specimens in different borehole arrangement schemes under the same high strain rate are shown (Figure 6). Compared with the results of previous studies pertaining to coal specimens under static tests, the compaction and closing stages of the dynamic stress–strain curves of the coal specimens under impact loading were not evident, mainly because of the shorter compaction and fissure closing times of the coal specimens owing to faster impact speeds. The dynamic stress curve exhibited softening characteristics, and was divisible into four stages: elastic; plastic; plastic energy dissipation; and failure. Compared to the intact coal specimen (N = 0), the stress–strain curve of the post-borehole coal specimens exhibited a local decrease in pre-peak stress, and the post-peak curve entered the plastic energy depletion platform. The main reason for the decrease in stress was that the stress concentration at the borehole edges under impact load accelerated crack extension around the borehole, causing the coal to collapse around the borehole. As shown (Figure 6), the stress drop characteristic of the single-borehole coal specimens (N = 1) was not obvious, on account of the smaller borehole diameter of single-borehole coal specimens, causing the range of collapse of the borehole to decrease, a result which was more consistent with those of previous studies [22]. The stress drops when the borehole diameter of the single-borehole marble specimens is > 6 mm; when n > 1 due to the number of boreholes increasing, the cracks around the boreholes become interconnected, the collapse range increases, and the range of decrease in the stress curves increases. As the borehole spacing increases, the range of the stress drop decreases, the stress drop of the coal specimens being the smallest at L = 3.5d. After the peak of the stress curve of the coal specimens, the stress curve of the borehole enters the platform of plastic dissipation. At this stage, the coal specimens have not yet lost their bearing capacity, and strain continues to increase after the peak, with the dissipation energy also increasing, causing the destruction of the coal specimens to lag. Overall, the stress before the peak decreases after the borehole, which reduces the accumulation of elastic strain energy before the peak is reached, and subsequent to the peak, the plastic platform increases in dissipative energy, thereby reducing the destruction of the brittle coal and lowering the destructive intensity of the rock burst.

### 3.2. Effects of Boreholes on Mechanical Properties

**Figure 7:**
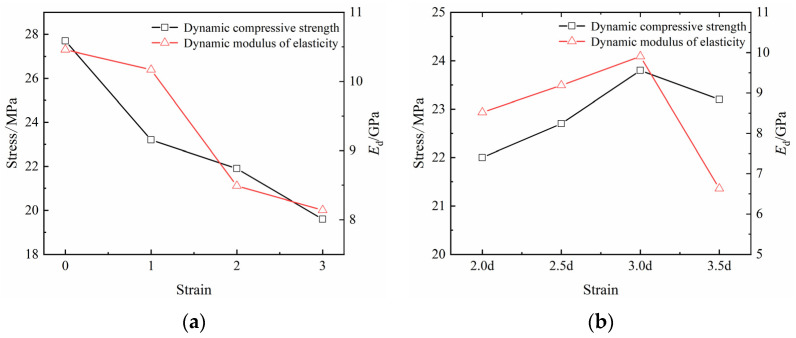
Peak stress variation diagrams of specimens with (**a**) different numbers of boreholes and (**b**) different spacings of boreholes.

The variation curves of the dynamic compressive strength and dynamic elastic modulus corresponding to different numbers of boreholes are shown (Figure 7a). The results showed that the dynamic compressive strengths of the coal specimens with N = 0–3 were 27.7, 23.2, 21.9, and 19.6 MPa, respectively, indicating that, compared to the complete coal specimens (N = 0), those with N = 1–3 showed decreases of 16.25, 20.94, and 29.24%, respectively. The dynamic moduli of elasticity (Ed) of coal specimens N = 0–3 were 10.46, 10.17, 8.49, 8.14 GPa, respectively, indicating that, compared to intact coal specimens N = 0, those with N = 1–3 showed decreases of 2.77, 18.83, and 22.18%, respectively. 

The variation curves of the dynamic compressive strength and dynamic elastic modulus at different borehole spacings are shown (Figure 7b); the dynamic compressive strengths of the coal specimens with borehole spacings of L = 2.0–3.5d were 22.0, 22.7, 23.8, and 23.2 MPa, respectively, indicating that the strengths decreased by 20.58, 18.05, 14.08, and 16.25%, respectively, compared with those of the complete coal specimens. The dynamic moduli of elasticity Ed were 8.52, 9.19, 9.91, and 6.63 GPa, and the decreases were 18.55, 12.14, 5.25, and 36.62% compared with the intact coal specimens, respectively.

The above findings indicate that under the effect of a high strain rate, the higher the number of boreholes, the lower the strength of the specimen, and the better the effect of pressure relief. The elastic modulus showed a decreasing trend, wherein the ability of the specimen to resist deformation decreased as the borehole spacing increased, and both the compressive strength and the elastic modulus of the coal specimens showed a trend of first increasing and then decreasing, showing that the boreholes may exert a certain deterioration and modification effect on the mechanical properties of the coal specimens.

### 3.3. Fracture Evolution

**Figure 8:**
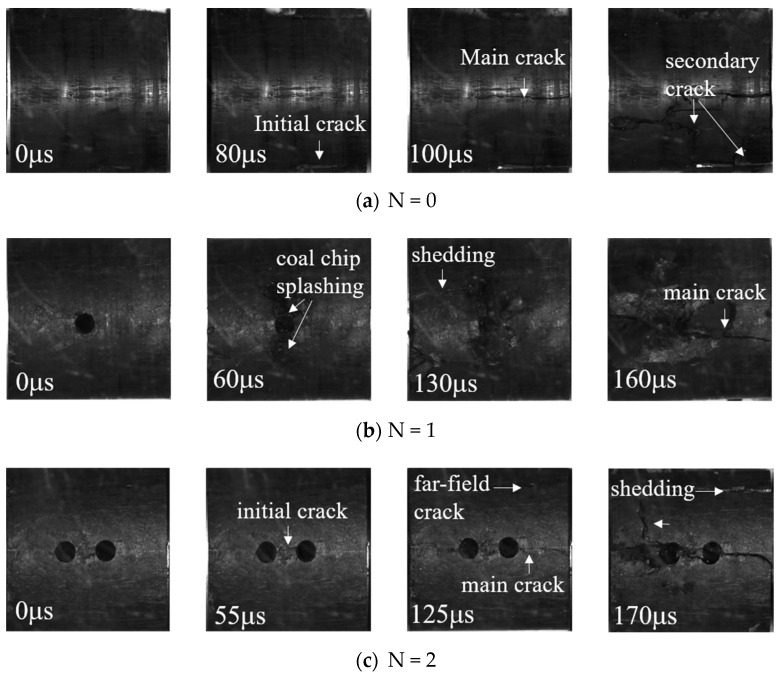

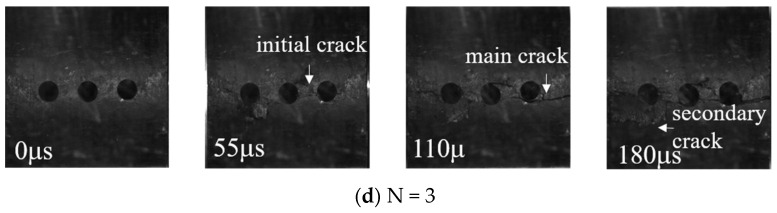
SEM images of crack propagation in specimens with different numbers of boreholes.

**Figure 9:**
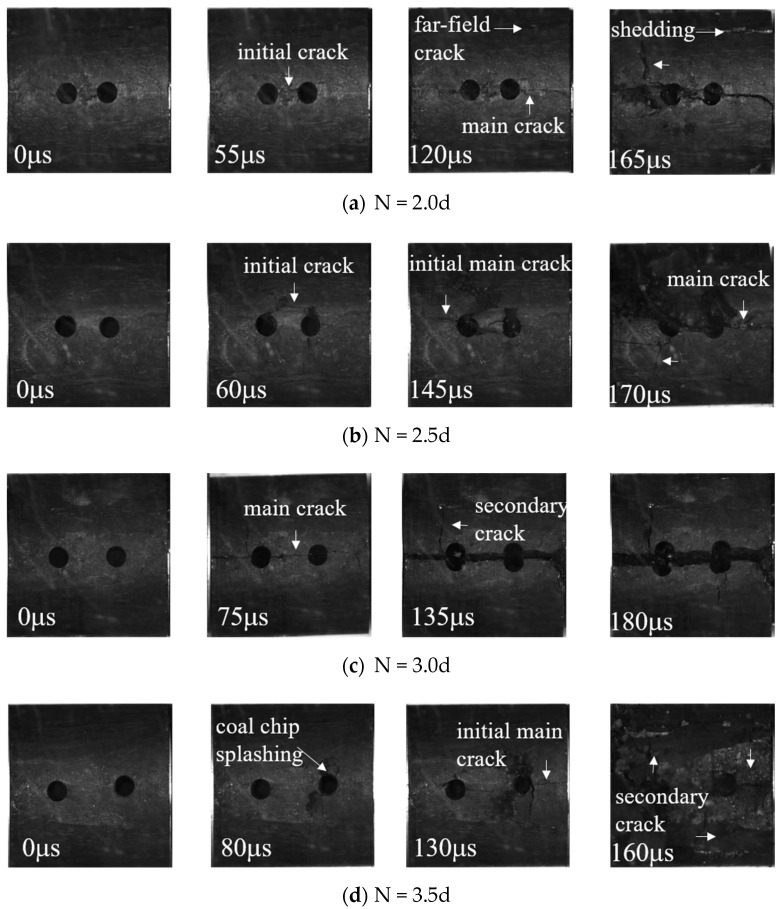
SEM images of crack propagation in specimens with different center spacings of boreholes.

A high-speed camera was used to photograph coal specimens with different borehole arrangements during the impact process [23,24,25]. The crack extensions and macroscopic damage of the coal specimens at different stages are shown (Figure 8 and Figure 9).

The crack expansion process of coal specimens with different numbers of boreholes are shown (Figure 8). At 80 μs, the initial cracks of the complete coal specimens were first generated below the coal specimens, with the main crack through the coal specimens being generated in the middle of the coal specimens along the direction of loading, following which secondary cracks sprouted perpendicularly to the main cracks.

At 60 μs, a coal chip splashing phenomenon similar to a rock burst was generated at the upper and lower edges of the single-borehole specimen; the cracks of the specimen continued to expand, leading to the surface coal body being partially dislodged. At t = 160 μs, the main cracks extended through the entire specimen via the borehole, in a direction parallel to the loading direction. The initial cracks of the two-borehole and three-borehole specimens were first generated between the borehole bridges of the boreholes, and as loading progressed, the main cracks expanded along the borehole bridges in a direction parallel to the loading direction, accompanied by the shedding of coal on the surface. The secondary cracks then sprang up and expanded in a direction perpendicular to that of the main cracks.

### 3.4. Fractal Features

Coal is a nonhomogeneous material containing pores and cracks, and macroscopic damage to coal results from the evolution of microscopic cracks, which are formed by the evolutionary convergence of smaller defects and cracks. This process, from the development of microscopic cracks to macroscopic destruction, shows fractal properties [26,27]. Currently, fractals are often used to show the self-similar characteristics of coal rocks.

(2) $$\frac{M_{\left(x\right)}}{M}={\left(\frac{A_{\left(x\right)}}{A}\right)}^{3-D}$$

where *M* is the total mass of the fragments, *M*_(*x*)_ is the cumulative quality under the aperture, *A*_(*x*)_ is the average grain size of the fragments, *A* is the maximum grain size of the fragments, and *D* is the fractal dimension of the fragment.

Taking the logarithms of both sides of Equation (2), we obtain the following:

(3) $$\mathrm{lg}(M_{\left(x\right)}/M)=(3-D)(\mathrm{lg}A_{\left(x\right)}/\mathrm{lg}A)$$

The slope *k* of the lg(*M*_(*x*)_/*M*)–lg*A*_(*x*)_/lg*A* curve may be obtained using Equation (3), and the relationship between the fractal dimension *D* and slope *k* is the following:

(4) D=3−k

**Figure 10:**
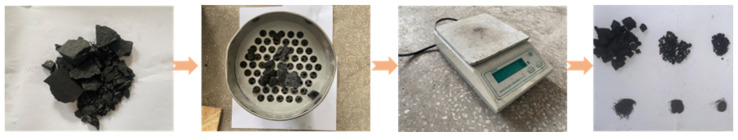
Schematic diagram of the screening process.

**Figure 11:**
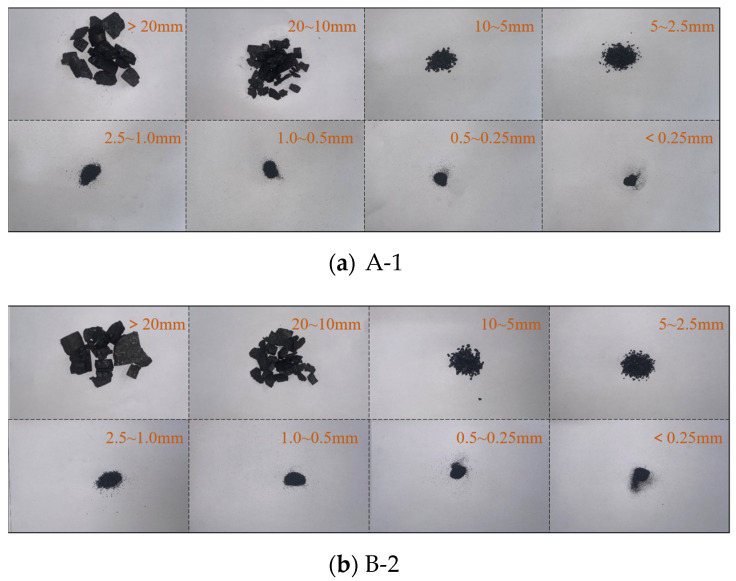
Dynamic failure morphology of some specimens with voids.

A schematic of the screening process is shown (Figure 10), and as shown in the figure, the crushed coal specimens were collected, screened, and weighed, following which eight groups of different ranks, namely, 0.00–0.25, 0.25–0.50, 0.5–1.0, 1.0–2.5, 2.5–5.0, 5.0–10.0, 10.0–20.0, and >20.0 mm, were screened. An electronic balance was used for weighing, and the screening results are shown (Table 1). The crushing-related morphology after screening is shown (Figure 11).

**Figure 12:**
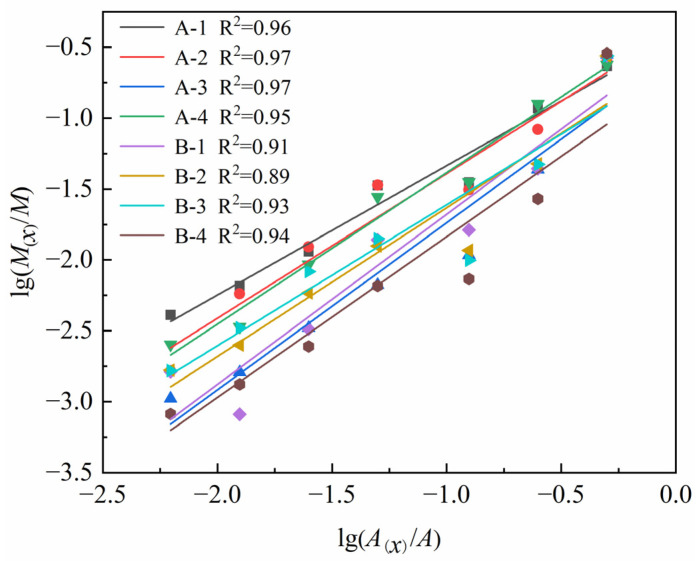
lg(*M*_(*x*)_/*M*)–lg(*A*_(*x*)_/lg*A*) curves in SHPB impact tests.

The lg(*M*_(*x*)_/*M*)–lg(*A*_(*x*)_/lg*A*) curves of coal specimens containing different numbers of boreholes and different spacings between the boreholes under high strain-rate loading are shown (Figure 12); the correlation between the scatter points of each group of data in the logarithmic curves of the fitted straight line is better. Adopting the intelligent method in the literature [28] for prediction, it was found that the block size of the coal specimens containing boreholes becomes very adaptive after impact damage and satisfies the fractal characteristics; the larger the fractal dimension, the greater the number of broken fragments, and the smaller the fragmentation, the larger the damage degree.

**Figure 13:**
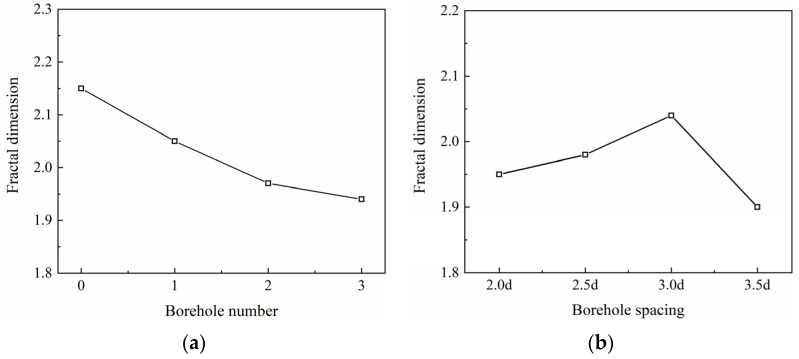
Relationship between the average fractal dimension and (**a**) different numbers of boreholes and (**b**) different spacing of boreholes.

The fractal dimensions (D) of the coal specimens with boreholes were calculated using Equations (2)–(4), and are listed in Table 1. According to the data in Table 1, it can be seen that under the action of constant high strain rate, with the increase in the number of drill holes, the particle size decreases, indicating that the degree of specimen crushing gradually increases, and it can be seen that the number of drill holes has a greater impact on the degree of destruction of coal samples after impact. The distribution of D with different numbers of boreholes and different spacings between boreholes (Figure 13) showed that the average fractal dimension exhibited a decreasing trend with increasing numbers of boreholes, 0–3, with the decreases being in the range of 4.65%, 8.83%, and 10.23%, respectively, compared to the intact specimens. The average fractal dimension shows an increasing and then decreasing trend with increasing borehole spacing (L), with the average fractal dimension being the largest when L = 3d, and the corresponding decreases being 9.30%, 7.90%, 5.11%, and 11.62%, respectively.

### 3.5. Energy Evolution

The evolutionary characteristics of energy are inevitably linked to fragmentation, and thus, the development and expansion of defects within coal rocks is essentially a process of energy dissipation. During the SHPB test, the incident energy (*W_I_*), reflected energy (*W_R_*), and transmitted energy (*W_T_*) were calculated, as follows:

(5) $$\left\{\begin{array}{l} W_{I}=\frac{A_{0}C_{0}}{E}\int_{0}^{t}\sigma_{I}(t)dt\\ W_{R}=\frac{A_{0}C_{0}}{E}\int_{0}^{t}\sigma_{R}(t)dt\\ W_{T}=\frac{A_{0}C_{0}}{E}\int_{0}^{t}\sigma_{T}(t)dt \end{array}\right.$$

where *σ_I_*(t), *σ_R_*(t), and *σ_T_*(t) are the stress–time curves of the corresponding incident, reflected, and transmitted waves, respectively.

During the impact test, the absorbed energy of the coal specimens mainly consisted of dissipative and ejection energies corresponding to the crushing of the coal specimen. The crushing dissipation energy was mainly spent on the generation of microcracks and crack expansion in the coal specimens, while the crushing kinetic energy was mainly spent on the ejection and splashing of broken pieces after the crushing of the coal specimens. The results showed that the crushing dissipation energy accounted for approximately 95% of the absorbed energy, while the crushing ejection energy accounted only for approximately 5% of the absorbed energy. Thus, the crushing kinetic energy is temporarily ignored, and the crushing dissipation energy equals the absorbed energy of the coal specimen, which is calculated as follows:

(6) $$W_{D}=W_{I}-W_{R}-W_{T}$$

The statistics of the incident energy, reflected energy, transmitted energy, and dissipated energy are shown (Table 2). These statistics indicate that compared with intact coal specimens, the transmitted energy of the coal specimens containing boreholes decreased while the reflected energy increased, showing that boreholes may effectively reflect stress waves, weaken the transmission ability of stress waves in the coal–rock medium under impact loading, and reduce the damage caused by stress waves to the surrounding rock.

Due to differences that exist between coal specimens, the values of the incident energy may differ under the same impact conditions. The reflected energy ratio (*W_R_/W_I_*), the transmitted energy ratio (*W_T_/W_I_*), and the dissipated energy ratio (*W_D_/W_I_*) are used to express the ratios of the reflected energy, transmitted energy, and dissipated energy to the incident energy, and to analyze trends in their energy changes.

**Figure 14:**
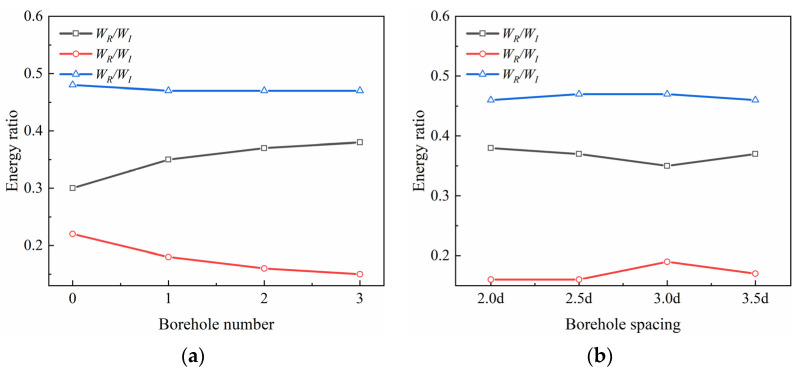
The relationship between the boreholes and the energy ratios for (**a**) different numbers of boreholes and (**b**) different spacings of boreholes.

Under the same impact, the reflected energy ratios of coal specimens with different numbers of boreholes show an increasing trend with increasing numbers of boreholes, while the transmitted energy ratios show a decreasing trend, and the overall trend of the dissipated energy ratio is not obvious (Figure 14). The reflective ability of coal specimens gradually increased with the number of boreholes, while the transmissive ability gradually decreased. The reflected energy ratios of coal specimens with different borehole spacings show a decreasing and then increasing trend with increasing borehole spacing (L), while the transmitted energy ratios show an increasing and then decreasing trend, whereas change in the dissipated energy ratio is not obvious. The curve indicates that for L ≤ 3d, the reflective ability of the coal specimens gradually increases with L, while their transmittance ability gradually weakens. When L > 3d, the reflective ability of the coal specimens weakens, while the transmissive ability gradually strengthens.

The dissipated energy of crushing refers mainly to the energy consumed by crack expansion in the coal specimens. Owing to the simultaneous transition of crack expansion in the coal specimens to the destruction of these specimens, as well as differences between individual coal specimens and errors in the kinetic energy of crushing and other energies, the overall trend in the change in dissipated energy becomes obscure. The coal around the borehole produces cracks under impact loading, and these cracks expand and penetrate each other, forming a crushing and pressure relief zone. This increases the reflection of the stress wave while simultaneously reducing the transmission of the stress wave, thereby reducing the damage caused to the surrounding rock by the impact stress wave under high strain-rate loading. The greater the number of boreholes, the larger the overall crushed zone formed by connected individual crushed zones, which can effectively enhance the ability to reflect the stress wave and reduce its transmission ability, resulting in the relief of pressure. Borehole spacing is also an important factor affecting pressure relief, where the smaller the borehole spacing, the greater the degree of fragmentation between boreholes, and the more obvious the effect of pressure relief. Therefore, a reasonable borehole spacing may help increase the effect of pressure relief, and also reduce labor and costs.

## 4. Discussion

Rock burst is recognized as an issue of global proportions in domestic and international mining and rock mechanics. Frequent roadway impact pressure-related accidents have caused numerous equipment losses and casualties, making the scientific effectiveness and applicability of measures, such as impact support and the unloading of impact pressure on roadway peripheral coal rock, a research hotspot in the field of impact pressure. Rock burst exhibits typical dynamic characteristics, and the propagation and attenuation of the impact stress wave produces an obvious high strain-rate loading effect on the peripheral rock of roadways. Therefore, the need to better understand impact prevention mechanisms and the quantitative design of unloading and support under high strain rates is urgent. Anchor net cables, grouting, and other supports play a role in the active reinforcement modification of the shallow peripheral rock of the roadway gang, which may be termed the “negative damage” modification effect. Weakening of the coal rock using boreholes artificially accelerates damage by forcing the peripheral rock into the limited equilibrium area, reducing the modulus of elasticity of coal rock, as well as the angle of internal friction and other basic mechanical properties; thus, the coal rock body veers in the direction of damage acceleration, which may be termed a “positive damage” modification effect. Therefore, the unloading of boreholes in tunnel rocks involves a typical positive damage modification principle for deep elastic surrounding rocks. This research mainly explored the positive damage modification effect of unloading boreholes, with the next step being in-depth research on the negative damage modification effect of the surrounding rock support, which connects the design of unloading and support for coal and rock bodies of the roadway to different damage levels, thereby helping to realize the design of “support-unloading” under multi-process parameters. 

### 4.1. Dynamic Mechanical Properties

**Figure 15:**
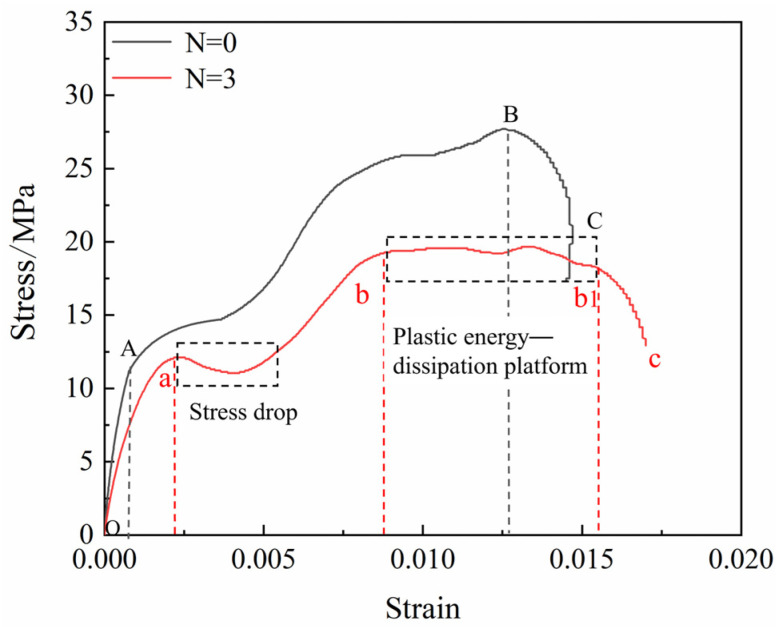
Stress–strain curves before and after boreholes.

The peak strength of the borehole coal specimens decreased under high strain-rate loading, while the impact reduction effect was enhanced. The dynamic stress–strain curves of the complete and borehole coal specimens are shown (Figure 15). The stress–strain curves of the complete coal specimens were divisible into three phases, as follows: the OA, elastic; the AB, plastic; and the BC, destructive. The borehole coal specimens increased the bb1 plastic energy dissipation stage compared to the intact coal specimens, and there was an evident stress drop phenomenon in the plastic stage, which was an important reason for the modification of the borehole at high strain rates.

**Figure 16:**
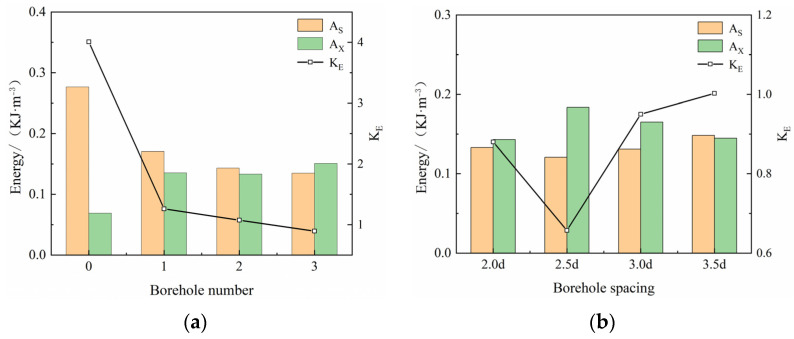
Variations in impact energy index of coal specimens with (**a**) different numbers of boreholes and (**b**) different spacings of boreholes.

Scholars usually judge impact propensity using impact propensity indices, such as compressive strength, dynamic failure time, elastic energy index, and impact energy index. The ratio of the deformation energy (A_S_), accumulated before the peak of the dynamic full stress–strain curve, to the dissipated energy (A_X_) after the peak, is termed the impact energy index (K_E_). K_E_ reflects the ability of the coal specimen to absorb and release energy. In this study, the dynamic impact energy index was chosen as the impact propensity criterion for drilled modified coal specimens under a high strain rate. The pre-peak accumulation energy decreased, and the post-peak dissipation energy increased after the borehole (Figure 16), and the dynamic impact energy index of the coal specimens was lower than that of the original complete coal specimens. A comparison between the complete coal specimens and those with an increased number of boreholes indicated that the dynamic impact energy index of the latter showed a linear decreasing trend, decreasing by 68.47%, 73.15%, and 77.66%, with the smallest KE of the coal specimens at N = 3. When the borehole spacing was increased, the dynamic impact energy index decreased and then increased, but decreased overall by 73.15%, 83.57%, 80.30%, and 77.34% for 2.0d–3.5d, respectively, with the smallest KE of the coal specimens at L = 2.5d. The decrease in stress caused by borehole collapse reduces the elastic deformation energy accumulated before the peak, and the increase in the plastic energy dissipation platform improves the post-peak dissipation energy of the coal rock. This indicates that the borehole of a coal body under a high strain rate exerts a certain deterioration effect on the impact energy index, reduces the impact propensity of the coal, significantly improves the effect of shock mitigation of the drilled coal rock, and reduces the degree of destruction of coal following the initiation of impact geo-pressure, which exerts a modifying effect on the coal.

The previous analysis indicated that the peak values and slopes of the stress–strain curves would vary with the numbers and spacings of boreholes under high strain-rate loading. The number and spacing of boreholes are important as well as sensitive parameters for coal modification. Due to limited space, this study only considered the modification effect exerted by the number and spacing of boreholes on coal rock. The next step will be to explore dynamic mechanical properties under parameters such as borehole diameter and inclination, and simultaneously quantify the equivalent relationship between borehole parameters as well as the design of coal seam borehole parameters.

### 4.2. Fracture and Energy Evolution

According to Section 3.3, the macro-damage form of the specimen manifested mainly as surface detachment and crack extension. Crack extensions caused by the development of internal joints in coal under the influence of the impact load on joints result in the dislodgement of coal pieces from the surface of the coal specimens, with such dislodgements showing a certain degree of randomness. In addition, the boreholes have a significant guiding effect on crack extension. The extended cracks in different borehole programs can be divided into A main cracks, B secondary cracks, and C far-field cracks. The main crack is a tensile crack produced by tensile stress, and its direction is parallel to the loading direction. The secondary crack is a fracture crack caused by extrusion, collision, and deformation between the coal itself and the compression bar during impact loading, and its direction is generally perpendicular to the main crack. The far-field crack, which is far away from the crack at the borehole, is related to the internal damage of the material. 

**Figure 17:**
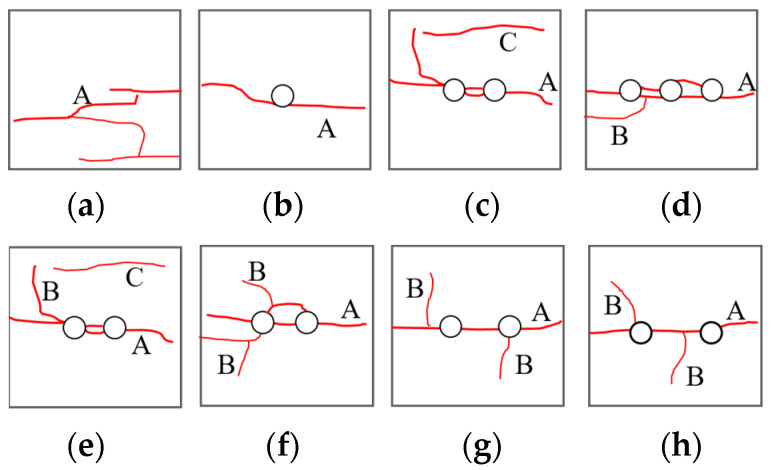
Sketch of cracks in borehole coal specimens with (**a**–**d**) different numbers of boreholes and (**e**–**h**) different spacings of boreholes.

**Figure 18:**
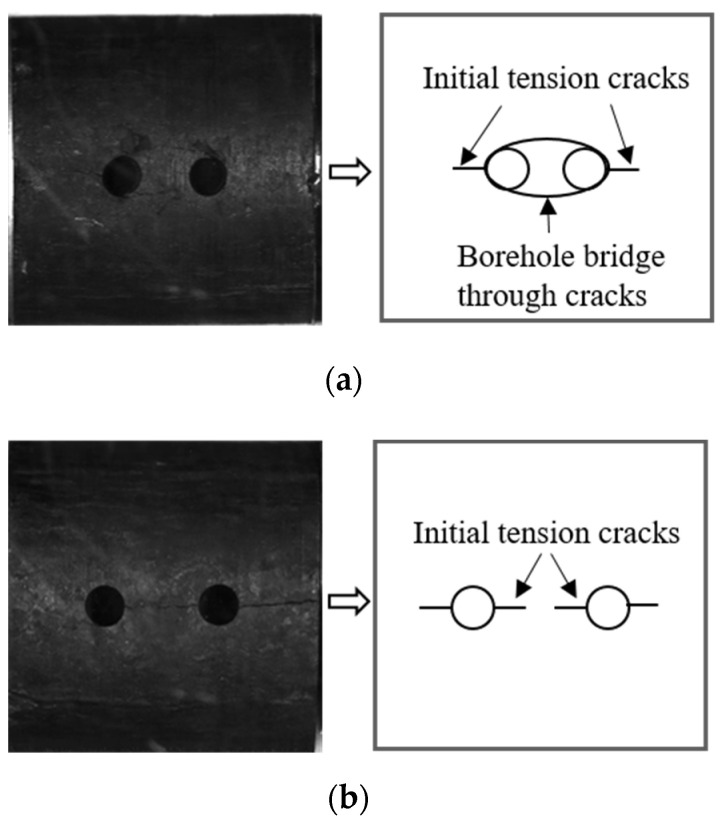
Crack formation patterns of borehole coal specimens under impact loading for (**a**) L = 2.0d and (**b**) L = 3.0d.

Under constant high strain rates, all coal specimens with different borehole arrangements undergo main and secondary cracks (Figure 17), which may act in unison to damage the specimens, with some coal specimens undergoing far-field cracks during the crack extension process. An analysis of the relationship between damage modes and different borehole spacings revealed differences between the crack penetration characteristics of the boreholes of the coal specimens. When the borehole spacing (L) exceeded 3d (L ≥ 3d), only one penetrating crack manifested between the boreholes. The larger spacings made the correlation between two boreholes weaker and the degree of independence stronger, leading to damage characteristics that were similar to those of a single borehole. When the borehole spacing was smaller (L < 3d), the correlation between two boreholes was stronger, and the number of cracks between the boreholes increased and extended through the two boreholes, indicating that the coal between the borehole bridges had been broken, allowing the two boreholes to form one whole borehole, which could be regarded as a new oval borehole, the spacing of which is shown (Figure 18).

The solution to the circular borehole problem in the plane strain of elastic mechanics indicated that, during the loading process of coal specimens, the two sides of the borehole edge parallel to the loading direction acted as tensile stress concentration areas. Therefore, the initial tensile cracks of the one-borehole coal specimens were generated on both sides of the borehole edge.

Section 3.2 indicates that under high strain rates, the boreholes exerted a significant guiding effect on crack extension in coal rock. Crack damage to intact coal rock mainly depends on the initial damage within the coal rock material. However, crack extension displays a certain randomness, the overall effect resulting in tensile damage to coal rock. The crack extensions of the coal specimens under different borehole arrangements were different, when compared with that of the intact coal rock. For example, the damage mode of multi-borehole coal specimens (N > 1) was different from that of single-borehole coal specimens (N = 1). Single-borehole coal specimens were damaged at the borehole edge by tensile damage due to impact loading, whereas multi-borehole coal specimens showed inter-borehole crushing zones where, when the borehole spacing was increased, the cracks between adjacent boreholes were transformed from penetrating cracks into independent cracks, and the degree of crushing between boreholes was reduced so that the decompression zones formed by the different borehole arrangements oriented in a manner that quantitatively increased the effect of shock decompression.

**Figure 19:**
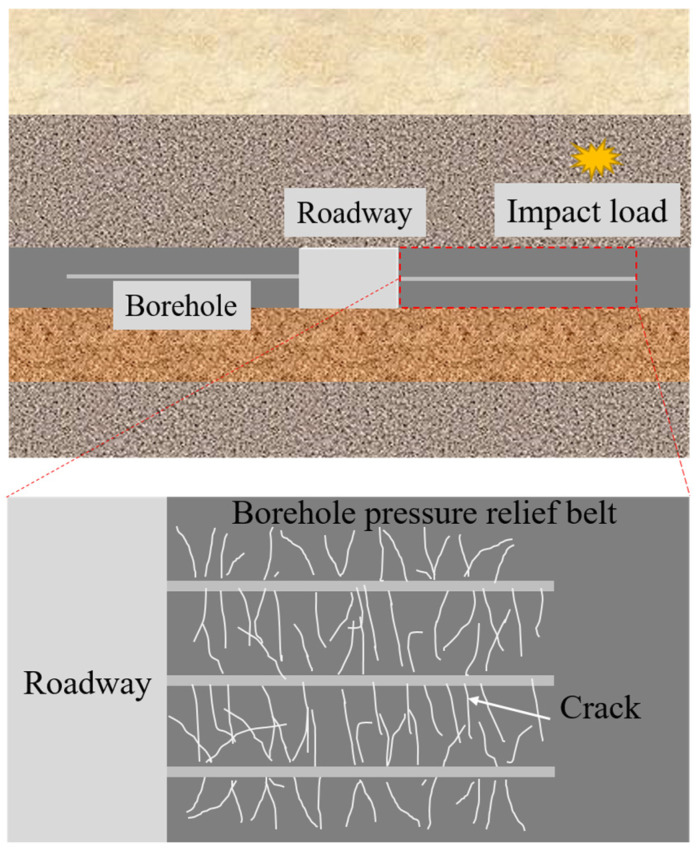
Unloading mechanism of perimeter rock boreholes under impact loading.

In deep mining, the dynamic disturbance generated following the initiation of impact pressure shows obvious dynamic mechanical characteristics in the surrounding coal rock under high strain-rate loading. After drilling, the crushed decompression zones become interconnected to form an overall decompression zone (Figure 19), which improves the reflective ability of the stress wave, but weakens its intensity, thereby reducing the destruction of coal rock following impact. Simultaneously, the collapse of the borehole provided a buffer space for the deformation generated during the rapid release of stress, reducing the degree of brittle damage to the coal rock after impact, and improving the stability of the surrounding rock.

**Figure 20:**
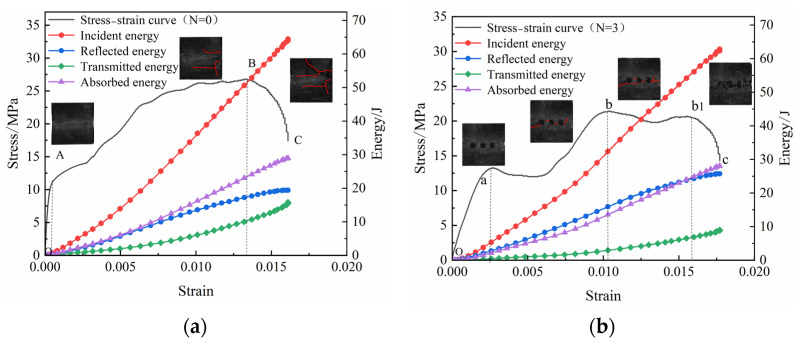
Energy evolution of intact coal specimens for (**a**) N = 0 and (**b**) N = 3.

Under constant high strain rates, the incident energy of coal specimens is mainly converted into reflected energy, transmitted energy, and absorbed energy (Figure 20), and the numerical changes in the different energies of the coal specimens before and after the drilling of the borehole following loading were as follows: absorbed energy > reflected energy > transmitted energy. However, in the OB stage, the reflected energy of the coal specimens of the drilled borehole > absorbed energy, and the growth rate of the reflected energy curve decreased when it was loaded to the B1 stage, and the curve of the transmitted energy was higher than that of the reflected energy at this time. The main reason for the curve change was that, after drilling, the expansion of cracks at the borehole edge formed a stress wave attenuation zone, which increased the stress wave reflecting ability of the coal body. When loaded to the b1c stage, the coal was damaged, and the energy used for crushing and absorption was increased.

In summary, boreholes may enhance the reflection of the stress wave in the plastic stage of coal while simultaneously reducing the transmission as well as the destructive intensity of the stress wave on the coal rock.

## 5. Conclusions

In this study, the SHPB test system was used to conduct dynamic impact tests on coal specimens subjected to different drilling programs, and to investigate the dynamic mechanical properties of coal specimens with different numbers and spacings of boreholes at a constant high strain rate. An ultrahigh-speed camera was used to record the damage process of the specimens and analyze the guidance laws of boreholes in the damage. The influence of boreholes on different energies and crushing characteristics was investigated. The following conclusions were drawn:

1. Boreholes deteriorate the mechanical properties of coal rock. As the number of boreholes increases, the dynamic compressive strength and dynamic modulus of elasticity decrease. The dynamic compressive strength and modulus of elasticity of the coal specimens tended to increase and then decrease with increasing hole spacing, indicating that the number and spacing of the boreholes are important sensitive parameters for impact modification.
2. Under impact loading, the initial tensile cracks of the drilled coal specimens sprouted in the tensile stress concentration area on both sides of the borehole edge, and then expanded into main cracks parallel to the loading direction; the specimen was damaged under the joint action of secondary cracks and far-field cracks. When the number of boreholes (N) exceeded 1 (N > 1), crushing occurred via multiple cracks penetrating between the boreholes, and when borehole spacing L exceeded 3.0d (L ≥ 3.0d), single cracks penetrated between the boreholes.
3. Under constant high strain rates, the fractal dimensions of the broken coal specimens showed a decreasing trend with increasing numbers of boreholes, 0–3; the rates of decrease were 4.65%, 8.83%, and 10.23%, respectively. The fractal dimension showed an increasing and then decreasing trend with increasing borehole spacings, 2.0–3.5d, and the ranges of decrease were 9.30%, 7.90%, 5.11%, and 11.62%, respectively. The incident, transmission, and dissipation energies of the borehole coal specimen decreased, while its reflective ability increased, and its transmission ability decreased with increasing numbers of boreholes and increased borehole spacing (L ≤ 3d).
4. This study investigated the equivalent relationship between the anti-punch drilling parameters and the coal rock-modified damage parameters under high strain-rate loading. The next step is to investigate the equivalent relationship between the anchorage support parameters and the damage parameters of coal rock modification under high strain-rate loading, and to establish the equivalent relationship between the coal rock damage variable, support parameter, and unloading parameter; and finally, to study the weakening control mechanism of the “support-unloading” partitioned coal rock modification on impact ground pressure under high strain-rate loading. The research results take the “support-unloading” equivalent damage modification parameter and its combination as an important parameter to weaken and control the impact ground pressure of surrounding rock, and lay a theoretical foundation for the quantitative design of the integration of roadway support and drilling unloading to prevent impact damage.

## Figures and Tables

**Table 1:** Particle size distribution and fractal dimension characteristics of porous specimen fragments.

Specimen No.	Mass of Debris between Sieves/g								D	$\bar{D}$	Correlation Coefficient
	>20 mm	20~10 mm	10~5 mm	5.0~2.5 mm	2.5~1.0 mm	1.0~0.5 mm	0.5~0.25 mm	<0.25 mm			
A-1-1	68.29	28.41	14.31	4.31	4.12	1.47	0.82	0.51	2.09	2.15	0.96
A-1-2	65.59	28.11	17.18	5.22	5.23	2.20	1.15	2.82	2.36		0.95
A-1-3	83.70	24.43	14.00	3.11	2.75	0.90	0.54	0.33	1.99		0.92
A-2-1	76.65	32.85	8.74	3.70	3.20	0.60	0.87	0.70	2.13	2.05	0.97
A-2-2	70.77	30.33	10.14	3.82	4.15	1.50	0.72	0.25	2.01		0.98
A-3-3	68.39	29.31	14.00	3.11	2.78	0.97	0.59	0.31	2.02		0.89
A-3-1	74.95	32.11	7.20	2.93	3.00	1.42	0.54	0.20	2.00	1.96	0.95
A-3-2	78.61	33.69	5.21	1.33	0.81	0.18	0.15	0.23	1.94		0.97
A-3-3	77.00	33.05	5.75	1.44	1.54	0.74	0.32	0.21	1.98		0.97
A-4-1	66.92	28.68	13.02	4.90	4.10	1.44	0.73	0.30	1.97	1.93	0.97
A-4-2	66.08	28.32	15.04	4.22	3.23	1.14	0.44	0.32	1.93		0.95
A-4-3	65.86	27.63	14.96	4.37	3.25	1.23	0.41	0.19	1.91		0.88
B-1-1	74.41	31.89	7.94	2.96	2.30	1.82	0.43	0.18	1.94	1.95	0.98
B-1-2	72.17	30.93	5.97	1.90	1.64	0.51	0.42	0.22	2.02		0.91
B-1-3	71.82	30.78	6.88	1.25	1.34	0.70	0.30	0.20	1.89		0.95
B-2-1	74.26	32.21	7.13	2.88	3.01	1.39	0.45	0.22	2.01	1.98	0.89
B-2-2	78.61	34.39	4.99	1.27	1.78	0.90	0.32	0.31	1.98		0.92
B-3-3	77.01	33.21	5.47	1.34	2.02	0.67	0.33	0.24	1.96		0.98
B-3-1	70.98	30.42	11.2	3.64	3.93	1.56	0.60	0.31	2.02	2.04	0.88
B-3-2	77.28	33.12	8.72	1.23	1.72	1.02	0.44	0.22	2.02		0.98
B-3-3	73.14	33.92	9.43	2.77	1.70	0.84	0.86	0.24	2.08		0.93
B-4-1	77.28	33.12	11.34	2.15	1.44	1.50	0.67	0.33	2.02	1.90	0.94
B-4-2	81.76	35.04	3.30	0.94	0.85	0.35	0.14	0.26	1.86		0.95
B-4-2	69.95	29.85	11.40	4.54	3.62	0.24	0.11	0.20	1.83		0.97

**Table 2:** Energy statistics of specimens.

Specimen No.	*W_I_*/J	*W_R_*/J	*W_T_*/J	*W_D_*/J	*W_R/_W_I_*	*W_T/_W_I_*	*W_D/_W_I_*
A-1	70.92	21.45	15.78	33.70	0.30	0.22	0.48
A-2	69.27	24.03	12.51	32.73	0.35	0.18	0.47
A-3	59.41	22.52	9.75	27.14	0.37	0.16	0.47
A-4	62.73	23.98	9.90	28.85	0.38	0.15	0.47
B-1	59.41	22.52	9.75	27.14	0.38	0.16	0.46
B-2	64.15	23.79	10.46	29.91	0.37	0.16	0.47
B-3	64.29	22.41	11.98	29.91	0.35	0.19	0.47
B-4	64.22	23.77	11.14	29.31	0.37	0.17	0.46

## Data Availability

The data presented in this study are available on request from the corresponding author.

## Abbreviations

SHPB	Split Hopkinson pressure bar
ISRM	International Society for Rock Mechanics
